# Transplant center assessment of the inequity in the kidney transplant process and outcomes for the Indigenous American patients

**DOI:** 10.1371/journal.pone.0207819

**Published:** 2018-11-21

**Authors:** Mira T. Keddis, Amit Sharma, Muneeb Ilyas, Nan Zhang, Hasan Khamash, Scott J. Leischow, Raymond L. Heilman

**Affiliations:** 1 Division of Nephrology and Hypertension, Department of Medicine, Mayo Clinic, Phoenix, Arizona, United States of America; 2 Division of Dermatology, Department of Medicine, Mayo Clinic, Phoenix Arizona, United States of America; 3 Department of Biostatistics, Mayo Clinic, Phoenix, Arizona, United States of America; 4 Office of Health Care Disparity, Mayo Clinic, Phoenix, Arizona, United States of America; University of Wisconsin, UNITED STATES

## Abstract

**Background:**

The goal is to determine the delays and reduced rates of kidney transplant (KTx) for the Indigenous Americans and variables predictive of these outcomes at a large single transplant center.

**Methods:**

300 Indigenous Americans and 300 non-Hispanic white American patients presenting for KTx evaluation from 2012–2016 were studied.

**Results:**

Compared to whites, the Indigenous Americans had the following: more diabetes, dialysis, physical limitation and worse socioeconomic characteristics(p<0.01); median difference of 20 day delay from referral to KTx evaluation, 17 day delay from approval to UNOS listing and 126.5 longer delay on the waitlist compared to whites(p<0.001). Of the Indigenous Americans listed, more died, were removed, or were still waiting than transplanted compared to whites (p<0.001). Variables predictive of delay from referral to transplant evaluation included: Indigenous race, distance from transplant center, coronary artery disease, and time on dialysis (p<0.05). Cumulative incidence of waitlisting and KTx was lower for Indigenous Americans (p<0.0001). Independent predictors of decreased likelihood of waitlisting included age, peripheral vascular disease, no caregiver, physical limitation, and illegal drug use history (p<0.05). Variables predictive of lower likelihood of KTx included Indigenous race, percentage of time inactive on the waitlist, no caregiver, and O blood type.

**Conclusions:**

Among patients referred and evaluated for KTx, the Indigenous American race was independently associated with significant delays in the KTx process after accounting for co-morbid and socioeconomic factors. Cardiovascular morbidity and physical limitation were identified as important determinants of delay and decreased likelihood of waitlisting. Further quantitative and qualitative work is needed to identify and intervene on modifiable barriers to improve access to KTx for the Indigenous Americans.

## Introduction

The Indigenous patients of the United States and across the world have been shown to have significantly increased burden of disease predisposing to end stage renal disease (ESRD) [1, 2]. While kidney transplant (KTx) remains the optimal treatment for ESRD for improvement in quality of life and overall survival, the Indigenous patients have lower annual rates of waitlisting and transplantation compared to whites [3–6]. In Australia, in addition to lower rates of waitlisting and KTx, the Indigenous Australians were shown to have higher cardiovascular risk and suffered poorer patient and graft survival especially among those with rural residence compared to non-Indigenous and urban residents [7]. Likewise, the Indigenous Canadians have lower rates of KTx compared to whites and these differences could not be explained by distance from the transplant center [8]. In the United States, cross-sectional studies from the early 1990s of dialysis patients showed similar transplant referral practices but significantly less likelihood for placement on the United Network for Organ Sharing (UNOS) waitlist and lower rates of KTx for the Indigenous Americans compared to whites [4–6]. In a study of hemodialysis patients from 1995 to 2006, annual rates of KTx were again shown to be lowest for the Indigenous Americans even compared to other minority groups and these differences were partly explained by clinical and socioeconomic factors [3]. These data have been derived from large patient registries such as the United States Renal Data System (USRDS) and UNOS and lack information on exact delays in the KTx process and an in-depth review of variables that may explain these delays. Moreover, while studies on African Americans and KTx access disparity have been increasing in the literature, similar and current studies for the Indigenous Americans are lacking.

The objective of this study is to assess the KTx process and outcomes and determine the contribution of co-morbid and socioeconomic factors that may explain the delays and the inferior rates of KTx among the Indigenous Americans at Mayo Clinic in Arizona. Arizona is part of ESRD Network 15 which is one of 18 networks funded by the United States government to monitor ESRD care. In Network 15, the Indigenous Americans account for 9.5% of prevalent dialysis patients, which is the highest percentage of Indigenous Americans on dialysis in the United States yet they represent only 5.1% of prevalent KTx patients [9]. Mayo Clinic Arizona transplant center is the largest transplant center with respect to transplant volume in Network 15 and ranks in the top 10 centers in KTx volumes nationwide therefore provides a robust platform for understanding differences in access to transplant for the Indigenous Americans that can be generalized to the larger transplant community [10].

## Materials and methods

The Mayo Clinic Institutional Review Board reviewed and approved this study (IRB 17–003369). Consent was waived as the study was deemed low risk by the IRB and the data were analyzed anonymously. Patients presenting for KTx evaluation between 2012 and 2016 at Mayo Clinic Arizona were reviewed (S1 File). Patients self-identified as American Indian, Native American or Alaskan Native in the medical record composed the Indigenous American cohort in our study. Because the majority of patients evaluated for KTx at our center are white Americans, we used a computer generated random sampling process to populate a comparison group of white non-Hispanic or Latino American patients matched for the year of transplant evaluation. The number of randomly selected white Americans matched the number of the Indigenous Americans per year of KTx evaluation. Matching for the year of transplant was necessary to account for transplant center practice related changes and changes in the KTx organ allocation process that took place during the time period of this study. Patients included in the study had to have had an initial KTx evaluation visit at our transplant center. A total of 300 Indigenous Americans underwent an initial KTx evaluation visit from 2012 to 2016. An equal number of white Americans was randomly selected during the same time period for a total of 600 patients.

### Outcome variables

There were three primary outcomes of interest for this study. First, KTx process delays which included the following time frames: time from referral to evaluation visit and time from evaluation visit to the following: selection conference committee decision, UNOS listing and transplantation. The second outcome of interest was KTx process outcomes which included the following rates: acceptance versus denial for transplant, waitlisting and outcomes after placement on the wait list. Reasons for deferral and denial were defined as cardiovascular, malignancy, infectious, physical limitation or psychosocial reasons. Psychosocial reasons included any of the following: failure to follow-up, lack of caregiver plan, lack of social support, drug abuse, or dialysis non-adherence (determined based on social worker investigation and assessment). The third outcome was to identify variables that explain delays in the KTx process.

### Patient variables

Patient variables of interest included those that encompass demographics, co-morbid conditions and socioeconomic variables that are known to be of clinical significance in assessing barriers to KTx and that may explain disparity in outcomes. The following were analyzed: age, gender, diabetes, history of heart failure, coronary artery disease, cerebrovascular disease, peripheral vascular disease, dialysis and modality and physical status. Coronary artery disease was defined as history of myocardial infarction, angioplasty, and/or coronary artery bypass surgery. Peripheral vascular disease was defined as requiring amputation or revascularization. Cerebrovascular disease was defined as either ischemic or hemorrhagic stroke. Physical limitation was defined as the inability to be independent with activities of daily living or requiring assistance with ambulation. This determination was based on the assessment of the evaluating transplant nephrologist and did not rely on an objective score. Socioeconomic variables included: caregiver support, substance use, smoking and alcohol history, marital status, annual income, education level, insurance type, employment status, and distance from the transplant center. The presence of caregiver support was determined by the social worker team who contacted the caregiver to confirm agreement to that role. Annual income data was extracted and poverty level income was assigned as $16,240 based on the U.S. Department of Health and Human Services poverty guidelines for a family of two. Distance to the transplant center was determined using zip code data.

### Statistical analysis

Continuous variables were presented as mean with standard deviation and as median with range for skewed distribution. Two sample t-test or Wilcoxon Rank Sum test was used for comparison of continuous variables between the two groups when appropriate. Categorical variables were summarized using counts and percentage and proportions were compared between the two groups by Chi-square test or Fisher’s exact test when applicable. Linear regression or logistic regression was used to examine the association between potential predictors and time between referral and evaluation, time between evaluation and determination of transplant candidacy and denial decision among patients who had final determination, time between approval and UNOS listing among patients who had been listed. Cumulative incidences of UNOS listing from the time of evaluation completion and the incidence of KTx from UNOS listing were estimated and compared between the two groups using Gray’s method in the presence of competing risk events (i.e., death or denial for transplant candidacy as competing risk events for UNOS listing and death or removal from the wait list was considered as competing risk events for KTx) [11]. A proportional hazards model for the sub-distribution of UNOS listing and KTx was used to estimate the hazard ratio for each potential predictor [12]. We examined each potential predictor in a univariate manner and clinically relevant factors that were statistically significant at 0.05 level were chosen into the final model. All statistical analyses were performed using SAS, version 9.3 (SAS Institute, Cary, NC).

## Results

**Fig 1:**
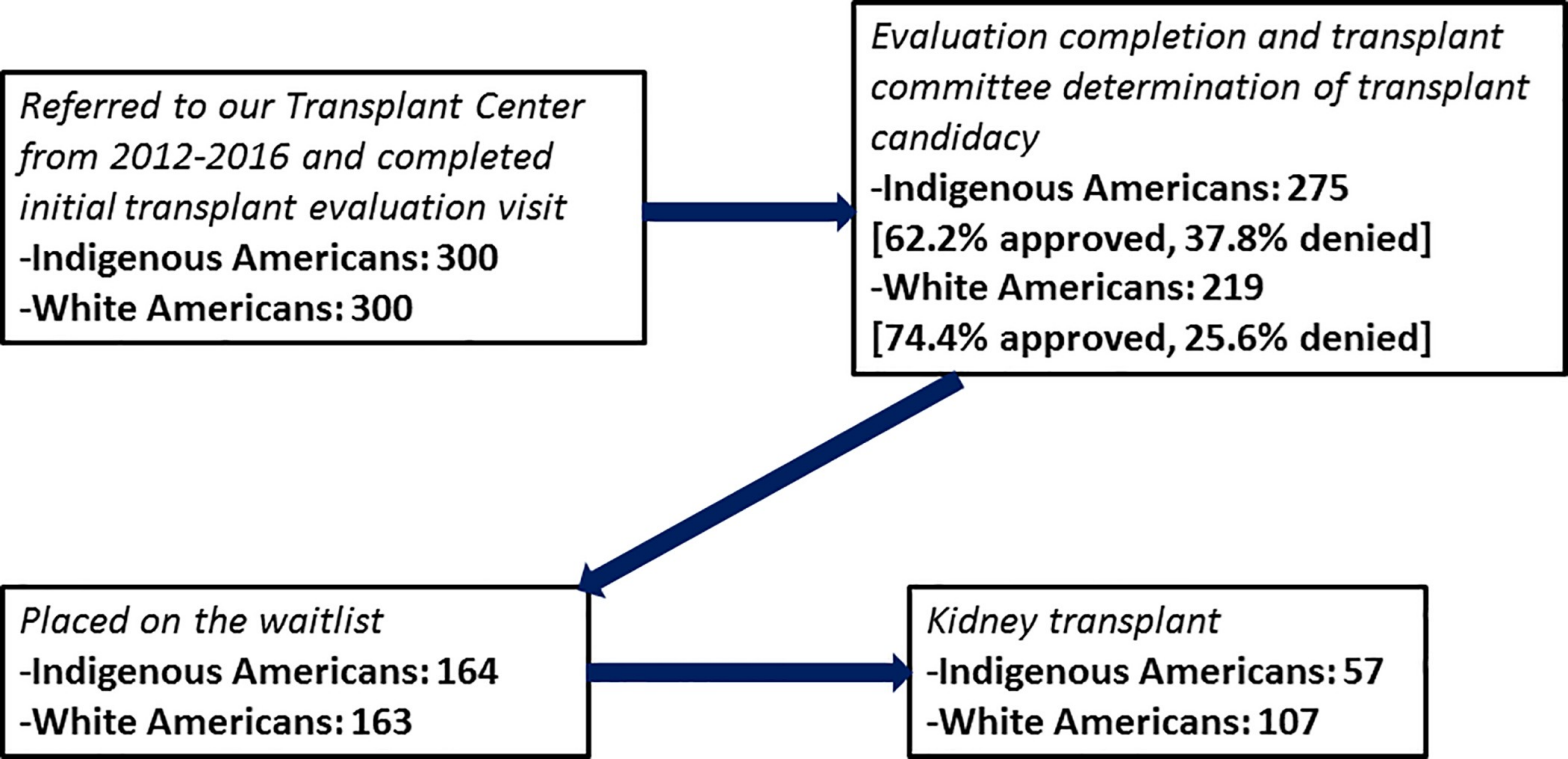
Flow chart of the outcome of the kidney transplant process for both groups.

All Indigenous Americans who presented for an initial KTx evaluation from 2012 to 2016 were studied (S2 File). Of the 600 patients who presented for KTx evaluation, 82.3% (n = 494) completed the KTx evaluation and were presented at the selection conference committee; of those 67.6% (n = 334) were approved and 32.4% (n = 160) were denied. Of those approved, 97.9% (n = 327) were listed and among those 50.2% (n = 164) were transplanted. Fig 1 shows the number of patients advancing through the KTx process between the two groups.

### Baseline characteristics

The mean age of the cohort studied was 55.1±13.0 years, 84% resided in the state of Arizona. Compared to whites, the Indigenous American patients were younger, more obese, had more physical limitation, were more likely to have diabetes and diabetic kidney disease, and require dialysis at the time of transplant evaluation. There was no significant difference in the prevalence of heart failure, coronary artery disease, cerebrovascular and peripheral vascular disease between the two groups as show in Table 1.

**Table 1:** Demographics and clinical risk factors for the Indigenous and white Americans.

	Indigenous (N = 300)	White (N = 300)	Total (N = 600)	p value^a^
Age at evaluation				<0.001
Mean (SD)	53.0 (12.2)	57.2 (13.4)	55.1 (13.0)	
Gender				0.934
Male	177 (59.0%)	178 (59.3%)	355 (59.2%)	
BMI Mean (SD)	30.8 (6.5), 295	29.2 (6.5), 298	30.0 (6.5), 593	0.003
				
Diabetes	237 (79.0%)	124 (41.3%)	361 (60.2%)	<0.001
Hypertension	235 (78.3%)	257 (85.7%)	492 (82.0%)	0.019
				
Heart failure	33 (11.0%)	23 (7.7%)	56 (9.3%)	0.161
Coronary artery disease	54 (18.0%)	63 (21.0%)	117 (19.5%)	0.354
Stroke	26 (8.7%)	18 (6.0%)	44 (7.3%)	0.210
Peripheral vascular disease	30 (10.0%)	27 (9.0%)	57 (9.5%)	0.676
Cancer	11 (3.7%)	60 (20.0%)	71 (11.8%)	<0.001
ESRD cause				<0.001
DM	200 (66.7%)	107 (35.7%)	307 (51.2%)	
Glomerulonephritis	39 (13.0%)	45 (15.0%)	84 (14.0%)	
Hypertensive/vascular	3 (1.0%)	27 (9.0%)	30 (5.0%)	
Polycystic kidney disease	1 (0.3%)	27 (9.0%)	28 (4.7%)	
Other	8 (2.7%)	37 (12.3%)	45 (7.5%)	
Unknown	21 (7.0%)	26 (8.7%)	47 (7.8%)	
Failed kidney transplant	28 (9.3%)	31 (10.3%)	59 (9.8%)	
Dialysis	264 (88.0%)	163 (54.5%), 299	427 (71.3%), 599	<0.001
Dialysis modality				0.011
Hemodialysis	229 (86.7%)	126 (77.3%)	355 (83.1%)	
Peritoneal	35 (13.3%)	37 (22.7%)	72 (16.9%)	
In-center vs home dialysis				<0.001
In-center	240 (93.8%)	109 (75.2%)	349 (87.0%)	
Home dialysis	16 (6.3%)	36 (24.8%)	52 (13.0%)	
Physical status				<0.001
No limitation	134 (44.7%)	231 (77.0%)	365 (60.8%)	
Any limitation	130 (43.3%)	50 (16.7%)	180 (30.0%)	
Severe limitation	36 (12.0%)	19 (6.3%)	55 (9.2%)	

^a^: Two sample t-test, Chi-square test or Fisher's exact test was used when applicable

### Socioeconomic characteristics

The Indigenous Americans had several socioeconomic characteristics that differed from whites presenting for KTx including: lower educational attainment, more likely to be single or widowed, and have higher prevalence of unemployment. The median annual income was lower than that of whites (median difference $ 31,518, p<0.001). The Indigenous Americans were more likely to have government based insurance while whites were more likely to have commercial insurance. The distance from place of residence to the transplant center was further for the Indigenous Americans than whites (see Table 2).

**Table 2:** Socioeconomic and psychosocial factors for the Indigenous and white Americans.

	Indigenous (N = 300)	White (N = 300)	Total (N = 600)	p value^a^
Education level				<0.001
Missing	15	3	18	
Less than high school education	72 (25.3%)	20 (6.7%)	92 (15.8%)	
High school education	131 (46.0%)	97 (32.7%)	228 (39.2%)	
Graduate school education	78 (27.4%)	144 (48.5%)	222 (38.1%)	
Post-graduate school education	4 (1.4%)	36 (12.1%)	40 (6.9%)	
Insurance				<0.001
Government-medicare	136 (45.3%)	44 (14.7%)	180 (30.0%)	
Government-medicaid	62 (20.7%)	18 (6.0%)	80 (13.3%)	
Medicare + private	63 (21.0%)	139 (46.3%)	202 (33.7%)	
Private	39 (13.0%)	99 (33.0%)	138 (23.0%)	
Marital status				0.001
Missing	3	1	4	
Married	131 (44.1%)	177 (59.2%)	308 (51.7%)	
With partner	30 (10.1%)	26 (8.7%)	56 (9.4%)	
Divorced	39 (13.1%)	42 (14.0%)	81 (13.6%)	
Widowed	23 (7.7%)	10 (3.3%)	33 (5.5%)	
Single	74 (24.9%)	44 (14.7%)	118 (19.8%)	
Unemployment	N = 297	N = 298	N = 595	
	244 (82.2%)	188 (63.1%)	432 (72.6%)	<0.001
Annual income				<0.001
Median (Range)	N = 272	N = 263	N = 535	
	15282 (0–160000)	46800 (0–720000)	26652 (0–720000)	
Annual income below poverty	N = 272	N = 263	N = 535	<0.001
	141 (51.8%)	49 (18.6%)	190 (35.5%)	
Caregiver	N = 292	N = 299	N = 591	0.116
	247 (84.6%)	266 (89.0%)	513 (86.8%)	
Smoking				0.133
Missing	1	0	1	
Never	147 (49.2%)	147 (49.0%)	294 (49.1%)	
Past	141 (47.2%)	131 (43.7%)	272 (45.4%)	
Current	11 (3.7%)	22 (7.3%)	33 (5.5%)	
Alcohol use				<0.0001
Missing	1	0	1	
Never	73 (24.4%)	113 (37.7%)	186 (31.1%)	
Past	221 (73.9%)	102 (34.3%)	324 (54.1%)	
Current	5 (1.7%)	84 (28.0%)	89 (14.9%)	
Illegal drug use				0.040
Missing	1	0	1	
Never	227 (75.9%)	244 (81.3%)	471 (78.6%)	
Past	64 (21.4%)	46 (15.3%)	110 (18.4%)	
Current	8 (2.7%)	10 (3.3%)	18 (3.0%)	
Distance from patient's home to transplant center (miles)				0.002
Median (Range)	112.1 (11.6–922.6)	44.2 (3.9–4989.7)	88 (3.9–4989.7)^b^	
Distance to transplant center				<0.001
≤ 88 miles	125 (41.7%)	188 (62.7%)	313 (52.2%)	
> 88 miles	175 (58.3%)	112 (37.3%)	287 (47.8%)	

a: Wilcoxon rank sum test, Chi-square test or Fisher's exact test was used when applicable

b: 88 miles is the median number of miles to transplant center for the entire cohort

### Kidney transplant process delays

The Indigenous Americans referred for KTx had longer delays compared to whites at every step of the KTx evaluation process with the most pronounced difference noted in waitlist time (median of 4 months longer wait time). Of those listed and transplanted, the median wait time from listing to transplantation was similar between the two groups (see Table 3).

**Table 3:** Differences in delays in the kidney transplant process between the two groups.

	Indigenous (N = 300)	White (N = 300)	Total (N = 600)	p value^a^
Time from referral to evaluation (days)	** **	** **	** **	**<0.001**
Median	75	55	66	
Range	(7.0–1703.0)	(0.0–1769.0)	(0.0–1769.0)	
Time from evaluation to initial decision (days) among patients who had decision	N = 275	N = 219	N = 494	<0.001
Median	34	22	28	
Range	(0.0–903.0)	(0.0–580.0)	(0.0–903.0)	
Time from evaluation to approval (days) among patients who had approval decision	N = 157	N = 152	N = 309	<0.001
Median	28	20.5	22	
Range	(6.0–637.0)	(4.0–295.0)	(4.0–637.0)	
Time from evaluation to deferral (days) among patients who had deferral decision	N = 35	N = 24	N = 59	0.057
Median	64	27	47	
Range	(7.0–384.0)	(6.0–356.0)	(6.0–384.0)	
Time from evaluation to denial (days) among patients who had denial decision	N = 83	N = 43	N = 126	0.212
Median	40	48	42.5	
Range	(0.0–903.0)	(0.0–580.0)	(0.0–903.0)	
Time from evaluation to approval in deferral group (days)	N = 14	N = 11	N = 25	0.002
Median	270	68	168	
Range	(56.0–749.0)	(22.0–263.0)	(22.0–749.0)	
Time from evaluation to denial in deferral group (days)	N = 21	N = 13	N = 34	0.035
Median	342	174	301	
Range	(109.0–617.0)	(6.0–720.0)	(6.0–720.0)	
Time from final approval to UNOS listing (days) among patients who have been finally approved and listed in UNOS	N = 164	N = 163	N = 327	<0.001
Median	31	14	20	
Range	(0.0–752.0)	(0.0–874.0)	(0.0–874.0)	
Time on UNOS list (days) among patients who have been listed in UNOS	N = 164	N = 163	N = 327	<0.001
Mean (SD)	703.8 (525.6)	474.3 (397.2)	589.4 (479.3)	
Median	545.5	419	468	
Range	(1.0–1965.0)	(3.0–1794.0)	(1.0–1965.0)	
Time from UNOS listing to Transplant (days) among patients who were transplanted	N = 57	N = 107	N = 164	0.360
Median	280	290	286	
Range	(1.0–1492.0)	(3.0–1651.0)	(1.0–1651.0)	

^a^: Two Sample t-test or Wilcoxon rank sum test was used when applicable

### Kidney transplant process outcomes

More Indigenous Americans were denied KTx than whites. The reasons for denial between the two groups were significantly different. The Indigenous Americans were more likely to be denied due to psychosocial or physical limitation while white Americans were more likely to be denied due to cardiovascular and malignancy causes. The median percentage time of active status in UNOS was significantly higher among whites compared to the Indigenous Americans. Patient outcomes after UNOS listing were different such that the Indigenous Americans were less likely to be transplanted, more likely to die on the wait list and be removed because of ineligibility compared to Whites (see Table 4).

**Table 4:** Kidney transplant process outcomes between the two groups.

	Indigenous (N = 300)	White (N = 300)	Total (N = 600)	p value^a^
Status after referral				
Had decision	275 (91.7%)	219 (73.0%)	494 (82.3%)	<0.001
Evaluation not complete	25 (8.3%)	23 (7.7%)	48 (8.0%)	
Lost to follow-up	0 (0.0%)	58 (19.3%)	58 (9.7%)	
Initial decision type among patients who had decision	N = 275	N = 219	N = 494	
Approval	157 (57.1%)	152 (69.4%)	309 (62.6%)	0.014
Deferral	35 (12.7%)	24 (11.0%)	59 (11.9%)	
Deny	83 (30.2%)	43 (19.6%)	126 (25.5%)	
Final decision type among patients who had decision	N = 275	N = 219	N = 494	
Approve	171 (62.2%)	163 (74.4%)	334 (67.6%)	0.004
Deny	104 (37.8%)	56 (25.6%)	160 (32.4%)	
Final reason of denial among patients who have been denied	N = 104	N = 56	N = 160	
Cardiovascular	19 (18.3%)	13 (23.2%)	32 (20.0%)	0.006
Malignancy	4 (3.8%)	12 (21.4%)	16 (10.0%)	
Infectious	2 (1.9%)	1 (1.8%)	3 (1.9%)	
Psychosocial	52 (50.0%)	18 (32.1%)	70 (43.8%)	
Functional status	27 (26.0%)	12 (21.4%)	39 (24.4%)	
Status after approval among patients who have been approved	N = 171	N = 163	N = 334	
Listed in UNOS	164 (95.9%)	163 (100.0%)	327 (97.9%)	0.031
Died before listed	4 (2.3%)	0 (0.0%)	4 (1.2%)	
Waiting to be listed	3 (1.8%)	0 (0.0%)	3 (0.9%)	
Percentage of time active on the waitlist among patients who have been listed in UNOS	N = 164	N = 163	N = 327	
Median	72.1%	99.1%	83.1%	0.002
Range	(0%-100%)	(0%-100%)	(0%-100%)	
Status after listing	N = 164	N = 163	N = 327	
On wait list	59 (36.0%)	32 (19.6%)	91 (27.8%)	<0.001
Transplanted	57 (34.8%)	107 (65.6%)	164 (50.2%)	
Died	17 (10.4%)	11 (6.7%)	28 (8.6%)	
Removed	31 (18.9%)	13 (8.0%)	44 (13.5%)	

^a^: Chi-square test or Fisher's exact test was used when applicable

### Determinants of delays

#### Variables predictive of the delay from the time of kidney transplant referral to evaluation

On multiple linear regression analysis of the cohort of 600 patients who initiated KTx evaluation, the Indigenous American race was associated with longer time from referral to evaluation compared to white race (35.1 days longer delay). Patients whose home distance was further than 88 miles from transplant center (88 miles was the median distance from the transplant center for the entire group) compared to those who resided less than or equal to 88 miles from the transplant center had 29 day longer delay and patients with coronary artery disease history compared to those without had 31.9 day longer delay, and those with increased time on dialysis at the time of evaluation had 3.8 day longer delay (per year on dialysis) from the time of referral to KTx evaluation (see Table 5).

**Table 5:** Multivariate linear regression analysis showing determinants of delays at various steps in the kidney transplant process.

**Variables predictive of delay from time of referral to evaluation**		
**Variable**	**Estimate (days)**	**p value**
Indigenous race	35.1 (12.0, 58.2)	0.003
Distance from transplant center greater than median of entire group (>88 miles)	29.0 (6.5, 51.6)	0.0116
Coronary artery disease	31.9 (4.1, 59.7)	0.0244
Time on dialysis per one year	3.8 (0.1, 7.5)	0.0438
**Variables predictive of delay from evaluation to determination of transplant candidacy**		
Indigenous race	35.8 (14.0, 57.6)	0.0014
Denial decision	56.6 (33.4, 79.8)	<0.0001
Below poverty level income	33.6 (10.1, 57.2)	0.0053
**Variable predictive of delay from approval to UNOS listing**		
Indigenous race	34.9 (8.1, 61.7)	0.011

#### Variables predictive of the delay from kidney transplant evaluation to determination of transplant candidacy

For patients who completed the transplant evaluation and were evaluated for candidacy (N = 494), multiple linear regression analysis showed that the Indigenous Americans had significantly longer delay from evaluation to determination of candidacy compared to whites; patients who received denial decision had much longer time from evaluation to decision compared to patients with approval decision, and patients with below poverty level income had longer delay compared to patients with above poverty level income (see Table 5). Variables predictive of denial decision on logistic regression analysis included older age (OR 1.02 per year, p = 0.041), absence of a caregiver (OR 2.80, p = 0.002), longer time on dialysis (OR 1.09 per year, p = 0.032), and presence of physical limitation (OR 1.90, p = 0.0122). Indigenous race was not an independent predictor of being denied KTx.

#### Variables predictive of the delay from approval to UNOS listing

Among patients approved for transplant (N = 334), two variables were predictive of delay in the time to UNOS listing: Indigenous race (44.7 days, p < .001) and government insurance (38.3 days, p = 0.003). On multiple linear regression analysis, only Indigenous race was significantly associated with delay to UNOS listing after approval (34.9 days, p = 0.011).

#### Cumulative incidence of waitlisting

**Fig 2:**
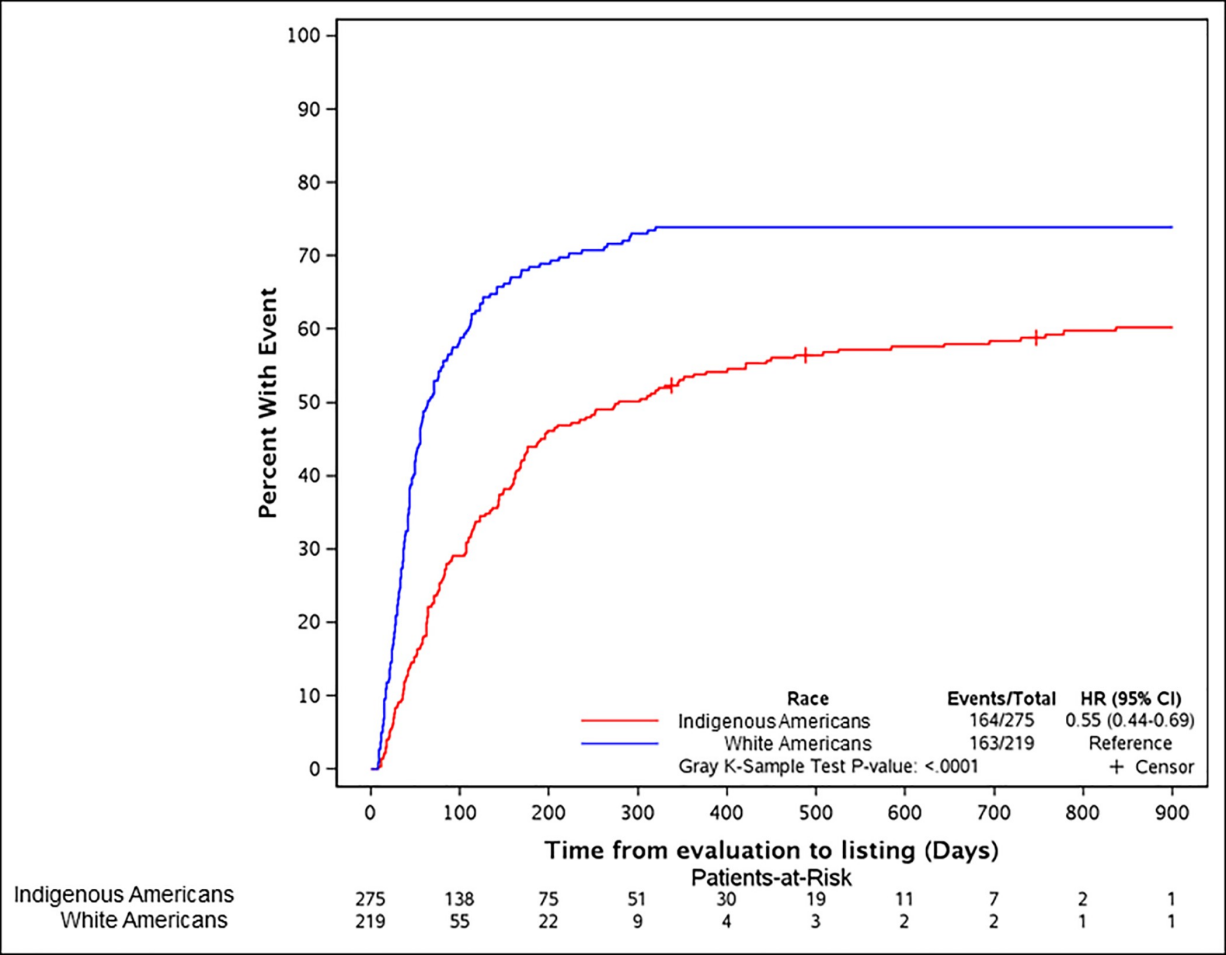
Incidence plot showing the inferior incidence of UNOS listing after completion of kidney transplant evaluation for the Indigenous Americans compared to whites.

Among patients who completed their transplant evaluation (N = 494), the cumulative incidence of waitlisting was 53.9% (48.2%, 60.1%) for the first year and 58.8% (53.2%, 65.0%) for the second year for the Indigenous Americans compared to 74.0% (68.3%, 80.1%) for the first and second years for whites (p<0.0001) (Fig 2). After accounting for denial decision by selection committee or death prior to waitlisting as competing risks, multivariate analysis showed the following variables to be predictive of lower likelihood of waitlisting: older age, physical limitation, and peripheral vascular disease, lack of a caregiver and any history of substance use (Table 6). Indigenous race was not a significant predictor.

**Table 6:** Univariate and multivariate time to event analysis for predicting the likelihood of placement on the UNOS waitlist after kidney transplant evaluation^a^.

Variables	Univariate HR (95% CI)	p value	Multivariate HR (95% CI)	p value
Age per year	0.99 (0.98, 1.00)	0.011	0.99 (0.98, 1.00)	0.0113
Time on dialysis per year	0.91 (0.86, 0.96)	0.0004	0.95 (0.90, 1.01)	0.0921
Indigenous race	0.55 (0.44, 0.69)	< .0001	0.80 (0.59, 1.08)	0.1422
Diabetes	0.63 (0.51, 0.79)	< .0001	1.00 (0.76, 1.33)	0.9728
Coronary artery disease	0.57 (0.43, 0.77)	0.0002	0.81 (0.58, 1.14)	0.2288
Peripheral vascular disease	0.43 (0.27, 0.68)	0.0004	0.54 (0.31, 0.93)	0.0266
Heart failure	0.48 (0.31, 0.75)	0.0012	0.66 (0.39, 1.12)	0.1228
Any physical limitation	0.57 (0.45, 0.72)	< .0001	0.66 (0.50, 0.87)	0.0031
Severe functional limitation	0.27 (0.16, 0.44)	< .0001	0.48 (0.27, 0.85)	0.012
Government insurance	0.55 (0.44, 0.69)	< .0001	0.89 (0.65, 1.22)	0.4735
Less than high school education	0.40 (0.23, 0.69)	0.0009	0.66 (0.37, 1.16)	0.1516
Below poverty level income	0.55 (0.43, 0.70)	< .0001	0.75 (0.55, 1.01)	0.0612
Absence of a caregiver	0.34 (0.22, 0.53)	< .0001	0.44 (0.27, 0.70)	0.0007
Current or past smoking	0.87 (0.70, 1.08)	0.2174		
Current or past alcohol use	0.92 (0.73, 1.16)	0.4736		
Current or past substance use	0.71 (0.54, 0.95)	0.0204	0.68 (0.49, 0.94)	0.0205
Distance from transplant center >88miles	1.13 (0.91, 1.40)	0.2713		

^a^The analysis is limited to patients who had final determination of transplant candidacy (n = 494)

#### Cumulative incidence of kidney transplantation

**Fig 3:**
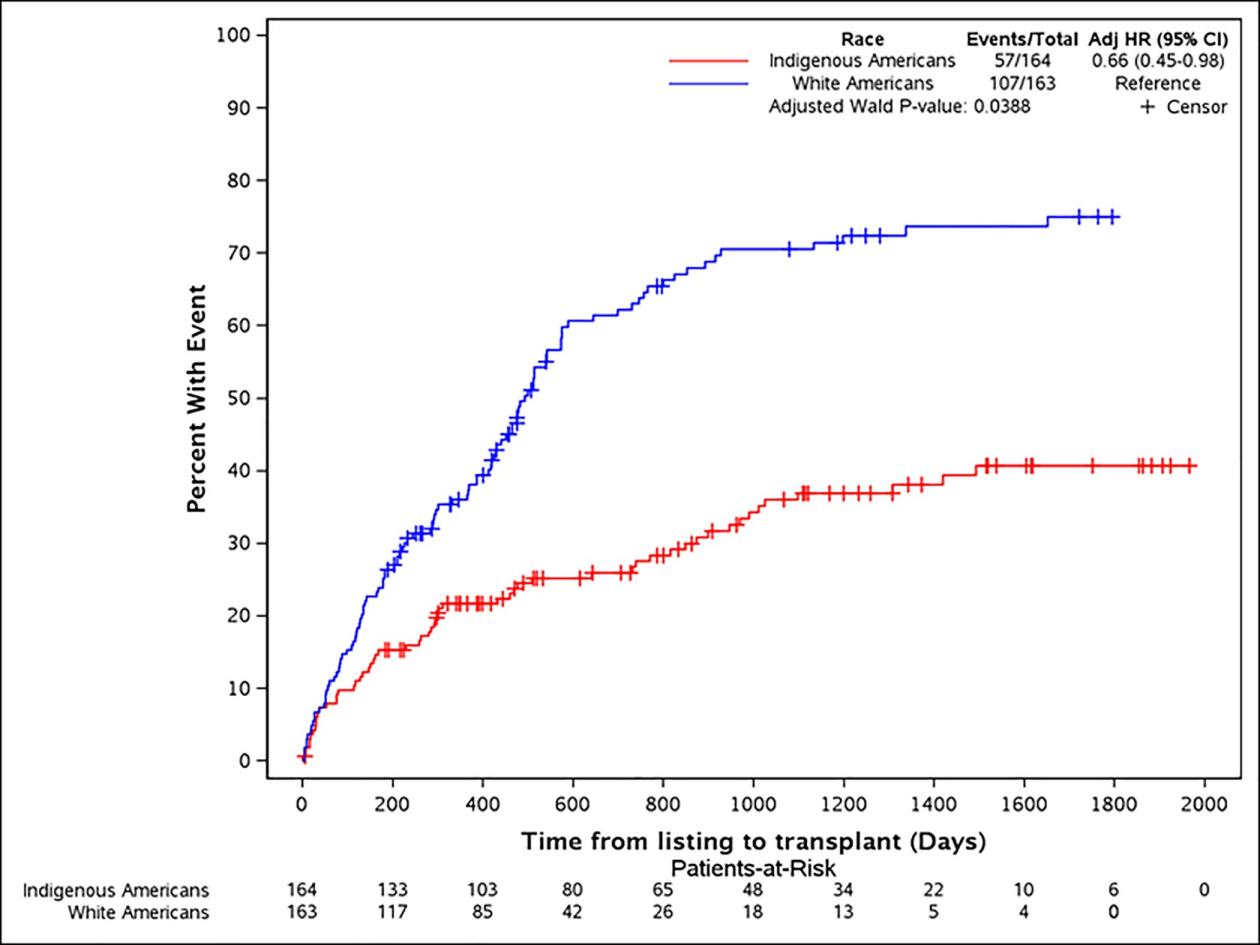
Incidence plot showing the inferior incidence of kidney transplants for the Indigenous Americans compared to whites.

Fifty seven (34.8%) Indigenous Americans versus 107 (65.6%) white Americans who were listed received a KTx. The cumulative incidence of KTx was significantly lower for the Indigenous Americans compared to whites (p<0.0001) (Fig 3). The percentage of living donation for the entire group was 28.7%. The Indigenous Americans were less likely to receive a living donor than whites (6 (14.3%) vs 35 (34.7%), p = 0.014). Table 7 summarizes the variables predictive of the likelihood of receiving a KTx after UNOS listing (N = 327). On multivariate analysis, O blood type, higher percentage of time inactive on the wait list, Indigenous race and lack of caregiver were the primary four variables predictive of lower likelihood of receiving a KTx.

**Table 7:** Univariate and multivariate time to event analysis for predicting the likelihood of kidney transplant after placement on the UNOS waitlist^a^.

Variables	Univariate HR (95% CI)	p value	Multivariate HR (95% CI)	p value
Age per year	1.00 (0.99, 1.01)	0.7991		
Time on dialysis per year	0.89 (0.83, 0.95)	0.0005	0.98 (0.92, 1.05)	0.5708
Indigenous race	0.39 (0.28, 0.54)	< .0001	0.66 (0.45, 0.98)	0.0388
Diabetes	0.57 (0.42, 0.77)	0.0003	0.87 (0.61, 1.23)	0.4311
Coronary artery disease	1.08 (0.70, 1.67)	0.7158		
Peripheral vascular disease	0.54 (0.25, 1.16)	0.1141		
Blood type O	0.50 (0.36, 0.68)	<0.0001	0.62 (0.45, 0.86)	0.0046
Heart failure	0.47 (0.20, 1.12)	0.0871		
Any physical limitation	0.61 (0.42, 0.88)	0.0086	0.99 (0.68, 1.45)	0.9647
Severe functional limitation	0.22 (0.07, 0.67)	0.0073	0.39 (0.11, 1.37)	0.1418
Active time on the waitlist (per 1% increase)	1.03 (1.02, 1.03)	< .0001	1.02 (1.02, 1.03)	< .0001
Government insurance	0.67 (0.49, 0.94)	0.0184	1.06 (0.74, 1.51)	0.7629
Absence of a caregiver	0.26 (0.10, 0.67)	0.0053	0.41 (0.17, 0.99)	0.0484
Distance from transplant center >88miles	0.64 (0.47, 0.87)	0.0045	0.73 (0.53, 1.01)	0.059

^a^The analysis is limited to patients who had been listed on UNOS (n = 327)

## Discussion

We show that the Indigenous American race, independent of other co-morbid or socioeconomic variables, was predictive of delays at many steps in the KTx process and was associated with decreased likelihood of receiving a transplant after listing. To our knowledge this is the first retrospective cohort study of the Indigenous American patients showing exact delays at every step of the KTx process and highlighting important determinants of these delays impacting all patients presenting for KTx evaluation.

Similar to our findings, Hispanic and Black patients were more likely to be on dialysis, less likely to be waitlisted after evaluation or receive KTx compared to white Americans [3] [6] [13]. Socioeconomic variables particularly government insurance and poverty level income were the most commonly cited predictors to explain delays to waitlisting [13–15] especially for the Black and Indigenous Americans [3]. Likewise, living donor transplantation has been shown to be lower for ethnic minorities (Hispanics, Asians, Black Americans) compared to white Americans [16]. Consistent with our findings, ABO blood type and not government insurance was a significant predictor to delay from waitlisting to KTx for Black and Indigenous Americans [3].

Despite younger age, 55.3% of the Indigenous Americans had physical limitation compared to 23% of whites. While this assessment was subjectively determined, it has important implications for understanding disparity in access to KTx as physical limitation was a significant determinant of delay in KTx evaluation completion, decreased likelihood of acceptance as a KTx candidate, and waitlisting. Physical impairment has been shown to be associated with older age, obesity, longer time on dialysis and diabetes [17]. Besides age, all these comorbid conditions have been shown to be more prevalent among the Indigenous Americans [3, 4, 18].

Socioeconomic factors contributed to 50% of the reasons for denial for the Indigenous Americans compared to 32.1% for whites. The contribution of poverty and insurance type to delays in KTx process was previously shown to partly explain the decreased rates of waitlisting for the Indigenous Americans and African Americans [3]. Consistent with this we show that government insurance and below poverty income both contributed to decreased likelihood of waitlisting. On multivariate analysis, these variables were no longer significant but older age, peripheral vascular disease, absence of a caregiver, the presence of any physical limitation and history of substance use were the primary determinants of delay in listing. Physical limitation is an indirect measure of frailty which has been shown to be associated with increased mortality and graft loss after transplant [19, 20]. The association of substance use with decreased likelihood of placement on the waitlist is consistent with other data showing lower rates of waitlisting and KTx and concerning implications after transplant [21–23]. The Indigenous American race was not independently predictive of the likelihood of progressing from evaluation completion to UNOS listing after censoring for those denied KTx and those who died prior to listing. Therefore by evaluating a select cohort of patients approved for KTx and who survived through the KTx process, race did not account for delays from the time of KTx evaluation to listing.

We hypothesize that the association of the Indigenous race with longer delay in Tx evaluation completion and presentation at selection committee may be explained by barriers in care coordination with the patients’ primary provider. Most Indigenous Americans are affiliated with a tribal health partner supported by the government. Optimizing care coordination between government funded tribal health services and specialty care such as transplant has been an ongoing quality effort [24]. The effect of lack of care coordination has been associated with disparity in KTx access for Black Americans [25].

The progression from approval to UNOS listing is a standardized process and our transplant center targets two weeks time period from the date of approval. Indigenous race was the only significant determinant of delay in timely listing on UNOS. We hypothesize that unmeasured confounders relating to establishing contact with the patient and the insurance provider at this step of the KTx process may account for the delay to listing.

The allocation process for transplant in the United States shifted the emphasis from wait time to dialysis time beginning in December of 2014. Since more Indigenous Americans required dialysis at the time of evaluation, in the cohort of patients who completed their transplant evaluation after December of 2014 (n = 103) the rates of approval for KTx and the median wait time on UNOS was similar between the groups. It is of note that the delays at every other step in the transplant process remained significantly longer with lower rates of KTx for the Indigenous Americans compared to whites.

The incidence of KTx among the Indigenous Americans has been shown to be the lowest compared to other minority groups [3]. Blood type O explained a significant proportion of the decreased rates of transplant for the Indigenous Americans compared to other groups [3] and our findings similarly show that the Indigenous Americans have higher percentage of blood type O (190 (70.4%) vs 118 (41.0%), p<0.001) and blood type O was associated with 40% reduction in the chance of receiving a KTx after UNOS listing.

We speculate that the Indigenous Americans’ perceptions and attitude about transplant may partly explain the independent association of the Indigenous race with 34% lower likelihood of receiving a KTx. A prior study of the Indigenous Americans showed that a significant proportion of patients who were deemed appropriate by the dialysis center for transplant referral did not complete the evaluation due to preference to remain on dialysis and not receive a KTx [6]. Qualitative evidence highlighted several barriers for KTx among the Indigenous Americans including mistrust of the safety of the deceased organ, ethical concerns about the procurement process, fear of surgery, and concerns about maintaining the body whole to maintain spiritual balance [26–29]. Similar barriers were identified among Black Americans including mistrust of the medical team, fear of surgery, care coordination barriers and other cultural factors (such as perceived discrimination) [25, 30–32]. Like the Indigenous Americans, the disparity in KTx access for Black Americans cannot be fully explained by medical or socioeconomic variables [3]. We hypothesize that barriers to KTx identified through qualitative work may explain the independent association of the Indigenous and Black races on inferior rates of progression in the KTx process. Future qualitative assessments to understand barriers to transplant for different racial groups at various steps in the KTx process are needed.

Several limitations warrant discussion. This is a single center study; however, we provide important co-morbid and socioeconomic variables that impact progression in the KTx process that should be evaluated for all KTx candidates regardless of race such as the KTx delays imposed by physical limitation, coronary artery disease, peripheral vascular disease and absence of dedicated support system. In addition, we hope that our findings spur similar studies in other transplant centers that serve the Indigenous populations to identify modifiable factors that can provide a culturally sensitive process for KTx. The time frames across each step of the KTx process are unique to our transplant center, but this does not change our conclusions regarding the differences noted between the Indigenous and white Americans. Racial and ethnic descriptions were ascertained from the medical record which can be imprecise and may create potential bias due to non-representative sampling, yet this would affect both groups studied. Lastly, our study did not take into account the effect of race on KTx referral. This has been previously evaluated in the United States and studies have shown similar referral rates to KTx among the Indigenous Americans compared to whites [4, 6].

### Conclusions

We have shown in this study that racial inequity in access to KTx for the Indigenous Americans persists in current times after accounting for a wide range of clinical, socioeconomic and psychosocial risk factors. These findings demand further qualitative and quantitative approaches to identify and address modifiable variables in an effort to eradicate racial inequity for the Indigenous Americans with kidney disease.

## Supporting information

S1 File The Mayo Clinic transplant center process. (PDF)

S2 File Deidentified data spreadsheet. (XLSX)
