# Supplementary material for: Transplant center assessment of the inequity in the kidney transplant process and outcomes for the Indigenous American patients
Source: PLoS One. 2018 Nov 21;13(11):e0207819. doi: 10.1371/journal.pone.0207819 (PMC6249016; doi:10.1371/journal.pone.0207819)
Supplement: S1 File — (PDF) [file pone.0207819.s001.pdf]

Transplant center process

The process for KTx evaluation at Mayo Clinic Arizona includes the following chronological steps: referral for KTx evaluation followed by authorization by the patient's insurance provider then a nurse directed phone questionnaire for screening for malignancy and cardiovascular disease. For patients with history of cardiovascular disease, reports of prior testing and intervention are requested for review prior to appointment. Once patients are deemed acceptable to proceed, a complete transplant evaluation process is scheduled including visits with transplant nephrology physician, social worker, dietician, financial services, surgery, education classes with a team of nurse coordinators and a wrap-up visit with a transplant provider, along with any additional evaluations deemed necessary after the initial visit. Patients are presented at the Selection Committee once evaluation is complete. Patients who are deemed acceptable candidates are listed on UNOS wait list once insurance authorization for listing is obtained for active listing (1-2 week process). Patients on the waitlist are required to return for annual testing and evaluation with the transplant nephrology team. Patients with incomplete workup who fail to present for their scheduled appointment will have their referral closed if the transplant center is unable to contact the candidate.
